# Autologous peripheral blood stem cell transplantation for Philadelphia chromosome‐positive acute lymphoblastic leukemia is safe but poses challenges for long‐term maintenance of molecular remission: Results of the Auto‐Ph17 study

**DOI:** 10.1002/jha2.677

**Published:** 2023-03-20

**Authors:** Satoshi Nishiwaki, Isamu Sugiura, Takahiko Sato, Miki Kobayashi, Masahide Osaki, Masashi Sawa, Yoshitaka Adachi, Motohito Okabe, Shigeki Saito, Takanobu Morishita, Akio Kohno, Takahiro Nishiyama, Hiroatsu Iida, Shingo Kurahashi, Yachiyo Kuwatsuka, Daisuke Sugiyama, Sachiko Ito, Hiroyoshi Nishikawa, Hitoshi Kiyoi

**Affiliations:** ^1^ Department of Advanced Medicine Nagoya University Hospital Nagoya Japan; ^2^ Division of Hematology and Oncology Toyohashi Municipal Hospital Toyohashi Japan; ^3^ Department of Immunology Nagoya University Graduate School of Medicine Nagoya Japan; ^4^ Department of Hematology and Oncology Nagoya University Graduate School of Medicine Nagoya Japan; ^5^ Department of Hematology and Oncology Japanese Red Cross Aichi Medical Center Nagoya Daini Hospital Nagoya Japan; ^6^ Department of Hematology Japanese Red Cross Aichi Medical Center Nagoya Daiichi Hospital Nagoya Japan; ^7^ Department of Hematology and Oncology Anjo Kosei Hospital Anjo Japan; ^8^ Department of Hematology and Oncology JA Aichi Konan Kosei Hospital Konan Japan; ^9^ Division of Hematology Ichinomiya Municipal Hospital Ichinomiya Japan; ^10^ Department of Hematology National Hospital Organization Nagoya Medical Center Nagoya Japan

**Keywords:** autologous peripheral blood stem cell transplantation, dasatinib, Philadelphia chromosome‐positive acute lymphoblastic leukemia, regulatory T cell

## Abstract

Autologous hematopoietic stem cell transplantation (SCT) is not a standard treatment option for Philadelphia chromosome‐positive acute lymphoblastic leukemia (Ph+ALL); however, its position has been reassessed since the introduction of tyrosine kinase inhibitors (TKIs). We prospectively analyzed the efficacy and safety of autologous peripheral blood SCT (auto‐PBSCT) for Ph+ALL patients aged between 55 and 70 years who had achieved complete molecular remission. Melphalan, cyclophosphamide, etoposide, and dexamethasone were used for conditioning. A total of 12 courses of maintenance therapy, including dasatinib, were performed. The required number of CD34^+^ cells was harvested in all five patients. No patient died within 100 days after auto‐PBSCT, and no unexpected serious adverse events were observed. Although 1‐year event‐free survival was 100%, hematological relapse was observed in three patients at a median of 801 days (range, 389–1088 days) after auto‐PBSCT. Molecular progressive disease was observed in the other two patients, although they maintained their first hematological remission at the last visit. Auto‐PBSCT can be safely performed for Ph+ALL with TKIs. A limitation of auto‐PBSCT was suggested, despite the increase in the intensity of a single treatment. The development of long‐term therapeutic strategies by including new molecular targeted drugs is warranted to maintain long‐term molecular remission.

## 1 INTRODUCTION

High‐dose chemotherapy followed by autologous hematopoietic stem cell transplantation (SCT) is a standard treatment for several hematological malignancies [1, 2]. Advances in testing techniques and treatments have led to using measurable residual disease (MRD) as a prognostic indicator for leukemia. As a result, the positioning of autologous SCT for acute leukemia has changed [3].

Allogeneic SCT (allo‐SCT) is a standard treatment option for Philadelphia chromosome‐positive acute lymphoblastic leukemia (Ph+ALL) [4], despite the dramatic improvement in the outcome of chemotherapy dramatically with the introduction of tyrosine kinase inhibitors (TKIs) [5]. In recent prospective studies, autologous SCT was performed in 19 and 35 patients using imatinib [6, 7]. Patient survival among those who underwent autologous SCT was equivalent to those who underwent allo‐SCT in both studies. Similarly, a large retrospective study, including 67 autologous SCTs, reported comparable results between autologous and allo‐SCTs [8].

Because autologous SCT has not been recommended in Japan since the introduction of TKIs, there is no data on autologous SCT for Japanese Ph+ALL patients. In this study, we planned to perform autologous SCT for Ph+ALL patients (aged 55 years or older) with complete molecular remission (CMR) who had transplant toxicity concerns for allo‐SCT, with the expectation of a more potent antileukemic effect than conventional chemotherapy.

## 2 PATIENTS AND METHODS

### 2.1 Study design

This was a multicenter prospective exploratory study with a target sample size of 5. The primary end point was the proportion of deaths within 100 days of autologous peripheral blood SCT (auto‐PBSCT). Secondary end points examining treatment efficacy included the proportion of molecular or hematological relapse, overall survival (OS), event‐free survival (EFS), molecular or hematological relapse rate, and non‐relapse mortality (NRM) at 100 days, 1 year, and 3 years after auto‐PBSCT. Regarding safety, the proportion of therapy‐related deaths, adverse events, engraftment failures, BCR‐ABL detections in harvested peripheral blood stem cells and BCR‐ABL mutation at relapse, the success rate of peripheral blood stem cell harvesting, and the ratio of the actual to the planned dose of dasatinib during maintenance were measured. De novo Ph+ALL patients aged between 55 and 70 years who achieved CMR within three chemotherapy regimens were registered in this study. The eligibility criteria have been reported elsewhere [9]. Survival and relapse were planned to be followed up for 3 years after auto‐PBSCT in the last enrolled patient. Adverse events were planned to be monitored until the end of maintenance therapy and evaluated according to the Common Terminology Criteria for Adverse Events (CTCAE) version 4.0. The study was conducted in accordance with the principles outlined in the Declaration of Helsinki, and all patients provided written, informed consent before registration. Patients were registered in this study after the data center in the Department of Advanced Medicine, Nagoya University Hospital, confirmed their eligibility. The protocol was approved by the institutional review board of each participating hospital. This study was registered in the UMIN Clinical Trials Registry with the identifier UMIN000026445.

### 2.2 Treatment schedule

The study protocol has been described in detail elsewhere [9]. Briefly, the study regimes consisted of three phases: peripheral blood stem cell harvest (PBSCH), conditioning prior to auto‐PBSCT, and maintenance (Table 1). Melphalan, cyclophosphamide, etoposide, and dexamethasone were used for conditioning. In maintenance therapy, dasatinib was planned to be administered for up to 60 weeks. In the first course of maintenance therapy, dasatinib was planned to be started at 50 mg/day, increased to 70 mg/day in the second week, and 100 mg/day in the third week. The dose of dasatinib was gradually reduced if grade 3 or higher hematological toxicity or grade 2 non‐hematological toxicity reappeared, even after recovery through drug cessation. In addition, when grade 3 or higher non‐hematological toxicity was observed, the dose of dasatinib was reduced in the same way (70 mg/day → 50 mg/day → 20 mg/day). After reducing the dose, it was not increased again, and the treatment continued at the reduced dose. MRD using RQ‐PCR for the detection of *BCR‐ABL1* chimeric gene was planned to be analyzed at designated time points: before conditioning for auto‐PBSCT, 30 days after auto‐PBSCT, before maintenance courses #4, #8, and #12, and 3 years after auto‐PBSCT.

**TABLE 1:** Auto‐Ph17 study regimens

Phase/drugs	Doses	Days
*PBSCH1*		
Cyclophosphamide	1200 mg/m^2^ (750 mg/m^2^ ^b^), IV	1
Daunorubicin	45 mg/m^2^ (40 mg/m^2^ ^b^), IV	1
Vincristine	1.3 mg/m^2^ ^a^ (1 mg/m^2^ ^a^, ^b^), IV	1
Prednisolone	60 mg/m^2^ (40 mg/m^2^ ^b^), PO	1–7
Methotrexate, dexamethasone	15 mg, 3.3 mg, IT	1
Dasatinib	100 mg/day, PO	PBSCH+1 to +15
*PBSCH2*		
Cyclophosphamide	1200 mg/m^2^ (750 mg/m^2^ ^b^), IV	1
Cytarabine	2 g/m^2^ (1 g/m^2^ ^c^, 500 mg/m^2^ ^b^), IV	2, 3
Etoposide	100 mg/m^2^, IV	1–3
Dexamethasone	40 mg/body, IV	1–3
Dasatinib	100 mg/day, IV	PBSCH+1 to +15
*Conditioning*		
Cyclophosphamide	60 mg/kg, IV	−4 to −3
Etoposide	500 mg/m^2^, IV	−4 to −2
Melphalan	130 mg/m^2^, IV	−1
Dexamethasone	40 mg/body (20 mg/body^b^), IV	−4 to −1
*M1*		
Dasatinib	50 mg/day, PO	1–7
Dasatinib	70 mg/day, PO	8–14
Dasatinib	100 mg/day, PO	15–28
*M2‐12 (every 5 weeks)*		
Vincristine	1.3 mg/m^2^ ^a^ (1 mg/m^2^ ^a^, ^b^), IV	1
Prednisolone	60 mg/m^2^ (40 mg/m^2^ ^b^), PO	1–7
Dasatinib	100 mg/day, PO	1–28

Abbreviations: IT, intrathecally; IV, intravenously; M, maintenance; PBSCH, peripheral blood stem cell harvest; PO, per os.

^a^ The maximum dose was 2 mg.

^b^ Dose for patients 65 years or older.

^c^Dose for patients aged between 60 and 64.

### 2.3 Immunological analysis of T cells

We assessed the numerical and phenotypic features of peripheral T cells at the same time as MRD analyses using multiparametric flow cytometry (FCM).

Peripheral blood mononuclear cells were isolated by density gradient with Ficoll‐Paque (GE HealthCare) and were stored in liquid nitrogen until analysis. Cells were washed with phosphate‐buffered saline containing 2% fetal calf serum (Biosera, Orange, CA, USA) and subjected to staining with surface antibodies, shown in Table S1. Intracellular staining of FOXP3 was performed with a monoclonal antibody against human‐FOXP3 and eBioscience Foxp3/Transcription Factor Staining Buffer Set (Thermo Fisher Scientific, Waltham, MA, USA) according to the manufacturer's instructions. After washing, cells were analyzed with a FACSymphony A3 (BD Bioscience, San Jose, CA, USA) and FlowJo version 10.6.1 (FlowJo LLC., Ashland, OR, USA).

### 2.4 Definitions

CMR was defined by the absence of detectable MRD with a sensitivity of at least 0.01%. Molecular relapse was defined when the *BCR‐ABL1* signal was detected after CMR was achieved. Molecular progressive disease was defined as an increase of 1‐log or more in the number of copies of *BCR‐ABL1* for a second consecutive time with an interval of at least 1 week. Hematological relapse was defined as (1) the appearance of leukemic blasts in peripheral blood, (2) 5% or more bone marrow blasts, or (3) the appearance of extramedullary lesions, after the hematological remission was achieved. Neutrophil engraftment was defined by an absolute neutrophil count of at least 0.5 × 10^9^/L for three consecutive days, and platelet engraftment was defined by a count of at least 20 × 10^9^/L without transfusion support.

### 2.5 Statistical analysis

The Kaplan–Meier method was used to estimate OS and EFS [10]. OS was measured from the date of auto‐PBSCT to the date of death from any cause, and surviving patients were censored at the date of the last contact. EFS was measured from the date of auto‐PBSCT until hematological relapse or death from any cause. The cumulative incidences of relapse and NRM were calculated using Gray's method [11, 12]. For relapse rate, death without relapse was the competing event; for NRM, relapse was the competing event. All statistical analyses were conducted with Stata version 17.0 software (StataCorp. LP, College Station, TX, USA) and EZR version 1.53 (Jichi Medical University Saitama Medical Center) [13], which is a graphical user interface for R (The R Foundation for Statistical Computing, version 4.1.0, Vienna, Austria).

## 3 RESULTS

### 3.1 Patient characteristics

**FIGURE 1:**
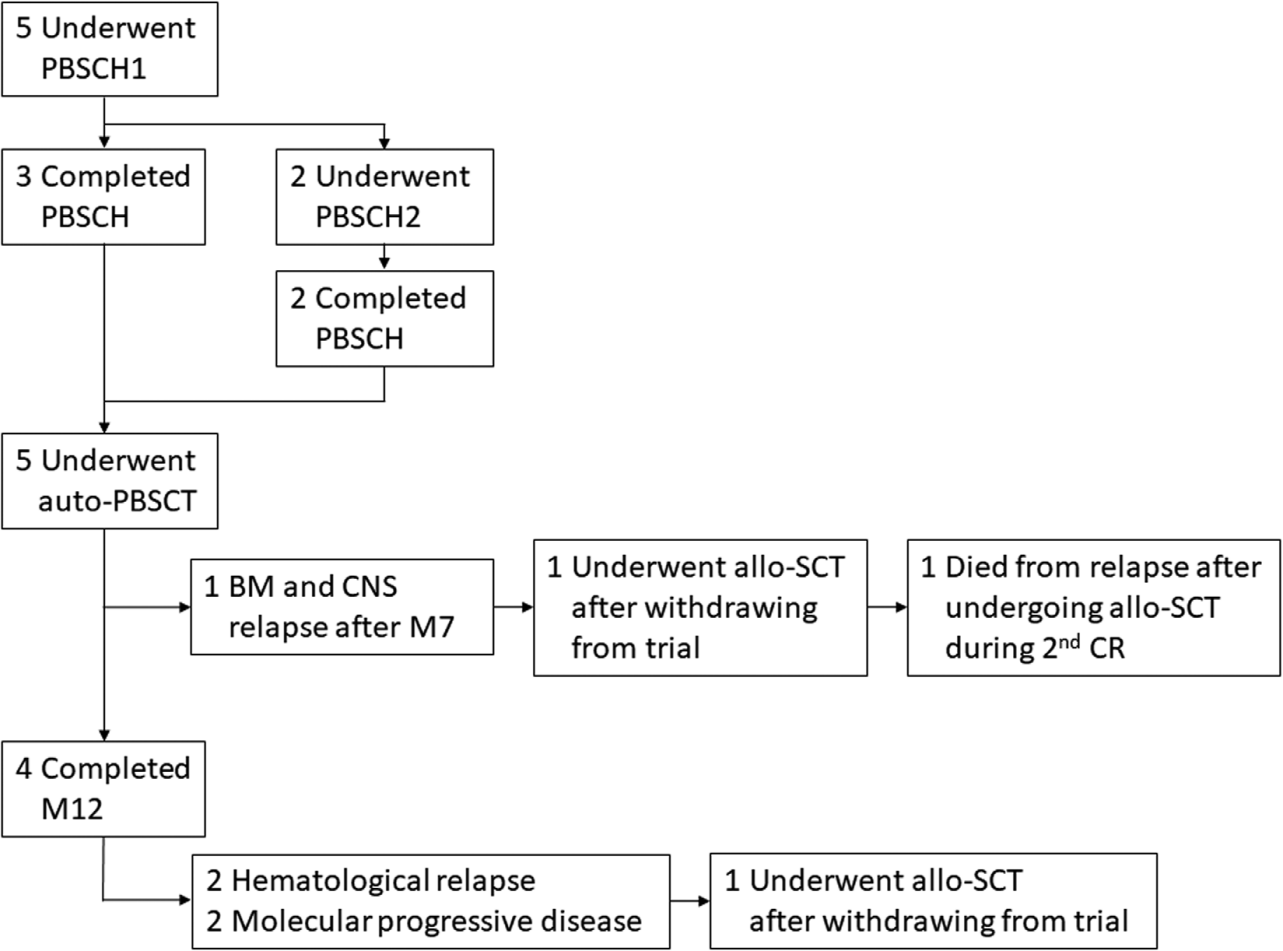
Patient flow and outcome. allo‐SCT, allogeneic stem cell transplantation; auto‐PBSCT, autologous peripheral blood stem cell transplantation; BM, bone marrow; CNS, central nervous system; CR, complete remission; PBSCH, peripheral blood stem cell harvest.

Between September 2017 and February 2019, five patients were enrolled in this study. The patient flowchart is shown in Figure 1. The median age at registration was 62 years (range, 59–68), the white blood cell count at diagnosis was 7100 (range, 600–168,700), and additional chromosomal abnormalities were identified in three patients (Table 2). CMR was achieved after one course of chemotherapy in three patients and after two courses of chemotherapy in two patients. The median time from diagnosis to CMR was 46 days (range, 43–76 days).

**TABLE 2:** Characteristics of patients and summary of peripheral blood stem cell harvest (PBSCH)

Patient ID	AP01	AP02	AP03	AP04	AP05
Age at registration	63	59	68	60	62
Sex	Male	Female	Female	Male	Female
PS at registration	0	0	2	0	0
WBC at diagnosis (/μL)	8600	168,700	600	7100	4300
PB blast%	57	96.4	5	50	3
BM blast%	86	88	65	61.5	80.6
CNS invasion at diagnosis	N	N	N	N	N
BCR subtype	Major	Minor	Minor	Minor	Minor
ACA	NA	+X, del(9p)	None	+8	+8, +X
No. of chemotherapy courses to reach CMR	2	1	1	2	1
Time from diagnosis to CMR (days)	76	45	46	56	43
*PBSCH*					
PBSCH1 CD34^+^ cells, ×10^6^/kg	3.85	0.99	2.00	0.78	1.91
PBSCH2 CD34^+^ cells, ×10^6^/kg	NA	3.66	NA	2.33	NA
PBSCH1 (days)	2	1	1	3	3
PBSCH2 (days)	NA	1	NA	1	NA

Abbreviations: ACA, additional chromosomal abnormality; BM, bone marrow; CMR, complete molecular response; CNS, central nervous system; NA, not applicable; PB, peripheral blood; PS, performance status, WBC, white blood cell.

### 3.2 PBSCH

**FIGURE 2:**
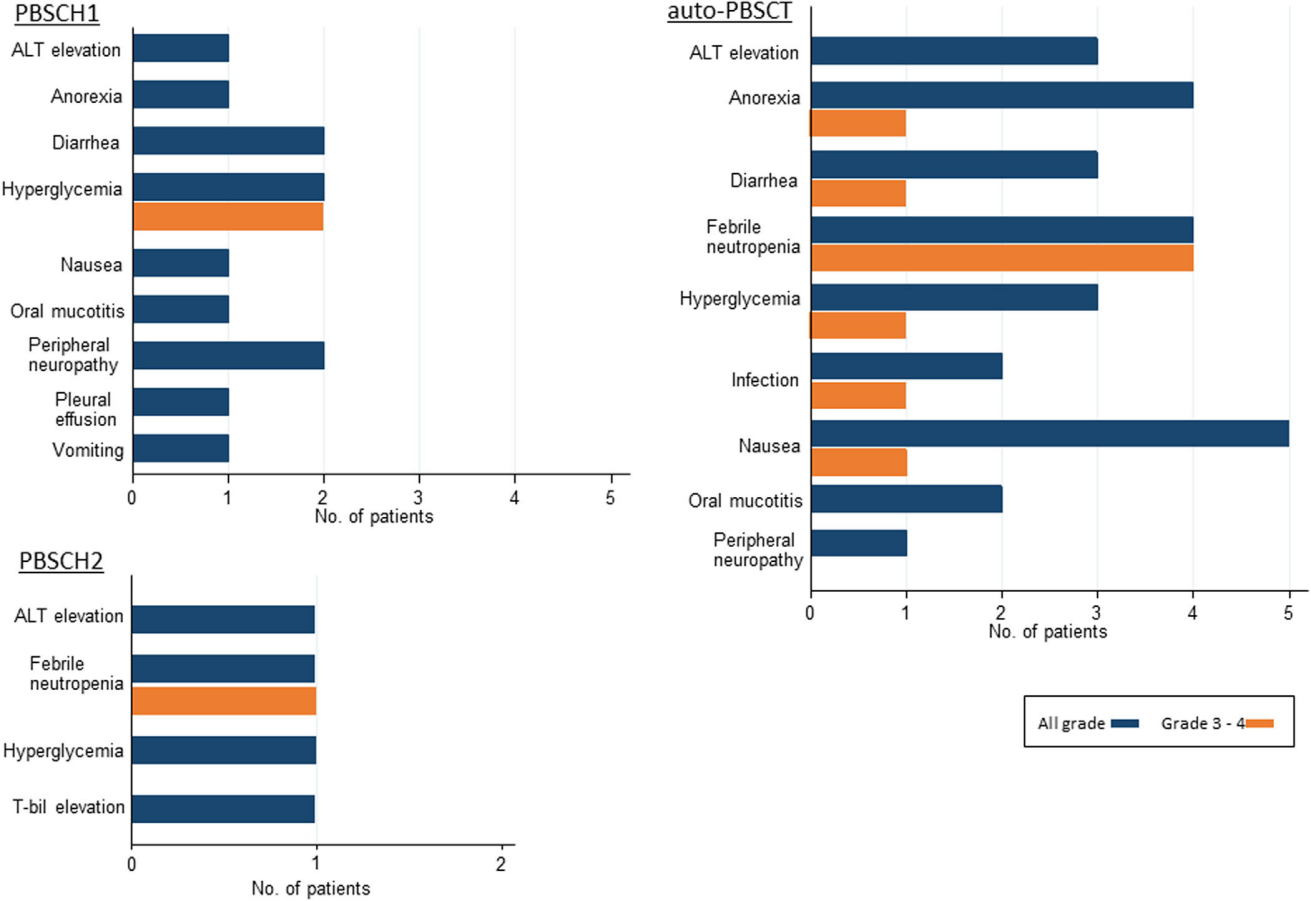
Non‐hematological adverse events during peripheral blood stem cell harvest (PBSCH) and autologous peripheral blood stem cell transplantation (auto‐PBSCT).

The target number of CD34^+^ cells (2 × 10^6^/kg) or more was harvested during the first PBSCH regimen in three patients, a median of 2 days of the procedure (range, 1–3 days). The median number of CD34^+^ cells was 1.91 × 10^6^/kg (range, 0.78–3.85 × 10^6^/kg) in all five patients. In the second PBSCH regimen, a sufficient number of CD34^+^ cells were harvested in both patients in 1 day of procedure using plerixafor. Most non‐hematological adverse events were grades 1 or 2, and no specific adverse events were observed in more than half of the patients (Figure 2).

### 3.3 Auto‐PBSCT

The median number of infused CD34^+^ cells was 3.03 × 10^6^/kg (range, 2.00–4.44 × 10^6^/kg). Neutrophil and platelet engraftments were achieved in all patients at a median of 10 days (range, 8–14 days) and 13 days (range, 8–21 days), respectively. No patient died up to 100 days after auto‐PBSCT. Febrile neutropenia was the most common severe adverse event (grades 3–4) during the auto‐PBSCT period (Figure 2). No unexpected serious adverse events were observed.

### 3.4 Maintenance therapy

**FIGURE 3:**
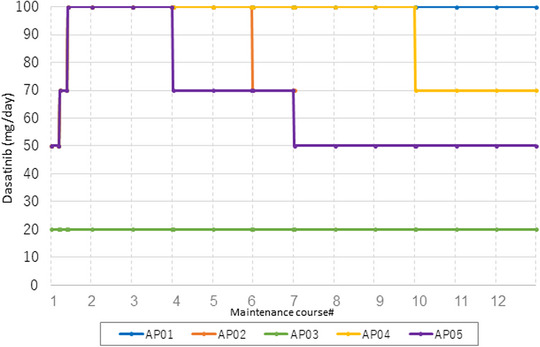
Doses of dasatinib during maintenance courses.

**FIGURE 4:**
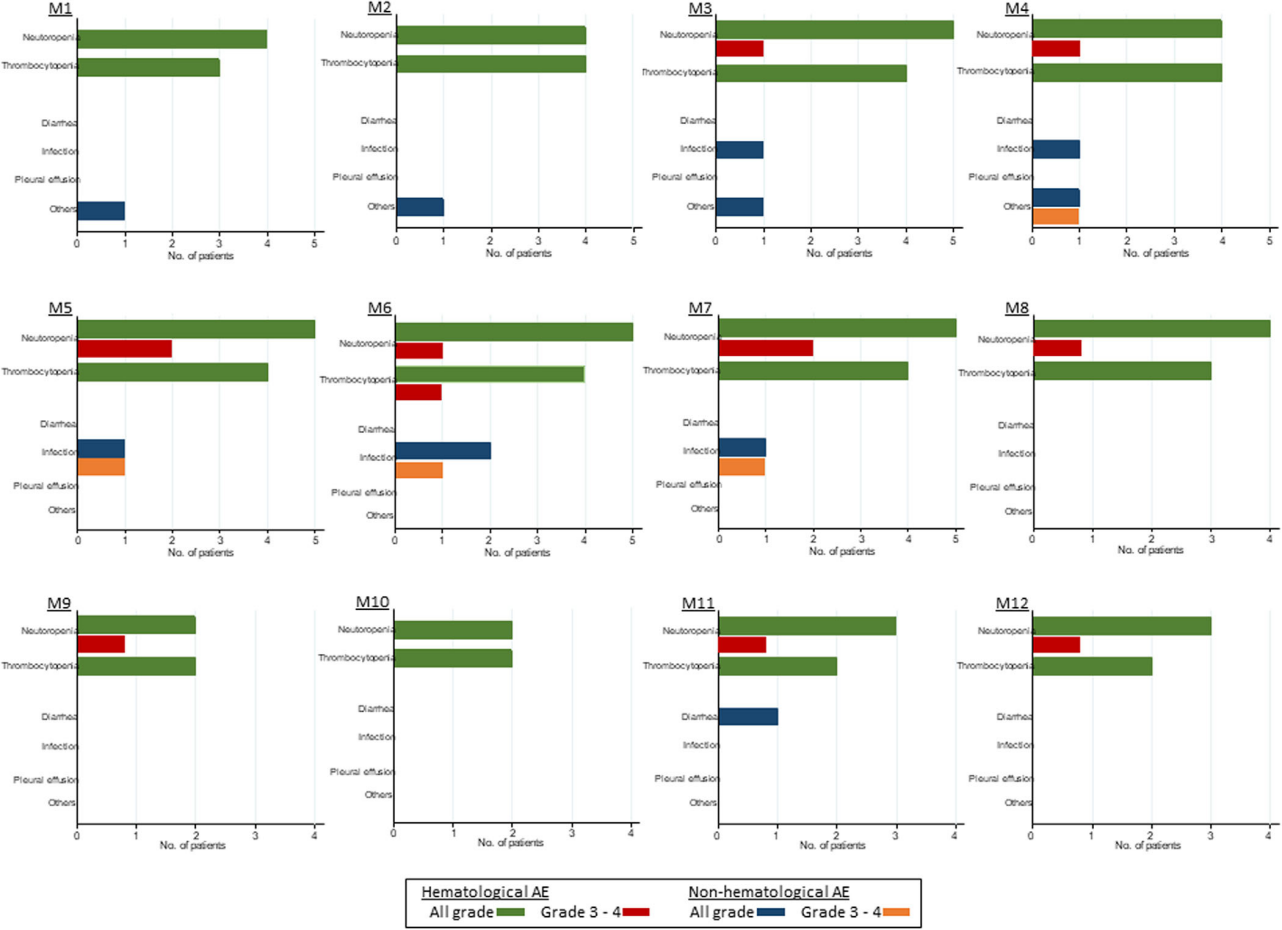
Adverse events during maintenance therapy. Each panel shows adverse events for each course of maintenance therapy.

Maintenance therapy was initiated at a median of 59 days after auto‐PBSCT (range, 30–69 days). All but one patient were able to start dasatinib at 50 mg/day as planned and gradually increased to 100 mg/day. Only 1 patient was able to receive dasatinib at 100 mg/day throughout the 12 courses; the other patients needed a dose reduction (Figure 3). Neutropenia and thrombocytopenia were frequently observed as hematological adverse events, but more than half of the cases were grades 1–2. On the other hand, non‐hematological adverse events were infrequently observed due to the appropriate dose reduction of dasatinib (Figure 4). All patients were able to continue dasatinib until the planned number of maintenance courses or relapse.

### 3.5 Long‐term outcomes

The median follow‐up period was 1100 days after auto‐PBSCT (range, 837–1519 days). The secondary end points are summarized in Table 3. Although 1‐year OS and EFS were 100%, a patient died of leukemia relapse 1051 days after auto‐PBSCT. The median survival time from diagnosis was 1310 days (range, 1107–1640 days). Hematological relapse was observed in three patients at a median of 801 days after auto‐PBSCT (range, 389–1088 days); however, all patients maintained their hematological remission for at least 1 year after auto‐PBSCT. ABL mutations were analyzed in two patients at relapse, but no T315I mutations were detected.

**TABLE 3:** Summary of the secondary end points

*Efficacy*	Day 100	1 year	3 year
Proportion of molecular relapse	0.2	0.2	1
Proportion of hematological relapse	0	0	0.6
OS (%)	100	100	75
EFS (%)	100	100	40
Molecular relapse rate (%)	20	20	100
Hematological relapse rate (%)	0	0	60
NRM (%)	0	0	0

*Safety*			
Proportion of therapy‐related death	0		
Proportion of adverse events	PBSCH	PBSCT	Maintenance
Hematological, all grade (grades 3–4)	1 (0.8)	1 (1)	1 (0.4)
Non‐hematological, all grade (grade 3–4)	1 (0.6)	1 (0.8)	0.6 (0.2)
Success rate of PBSCH			
Overall		100%	
PBSCH1		60%	
Detection of BCR‐ABL in harvested PBSC	0 (0/1)	0 (0/1)	
Proportion of engraftment failure		0	
Ratio of actual dose of dasatinib to planned dose during maintenance therapy, median (range)		0.91(0.2‐1)	
Mutation analysis of BCR‐ABL at relapse		0(0/2)	

Abbreviations: EFS, event‐free survival; NRM, non‐relapse mortality; OS, overall survival; PBSC, peripheral blood stem cell; PBSCH, peripheral blood stem cell harvest; PBSCT, peripheral blood stem cell transplantation.

**FIGURE 5:**
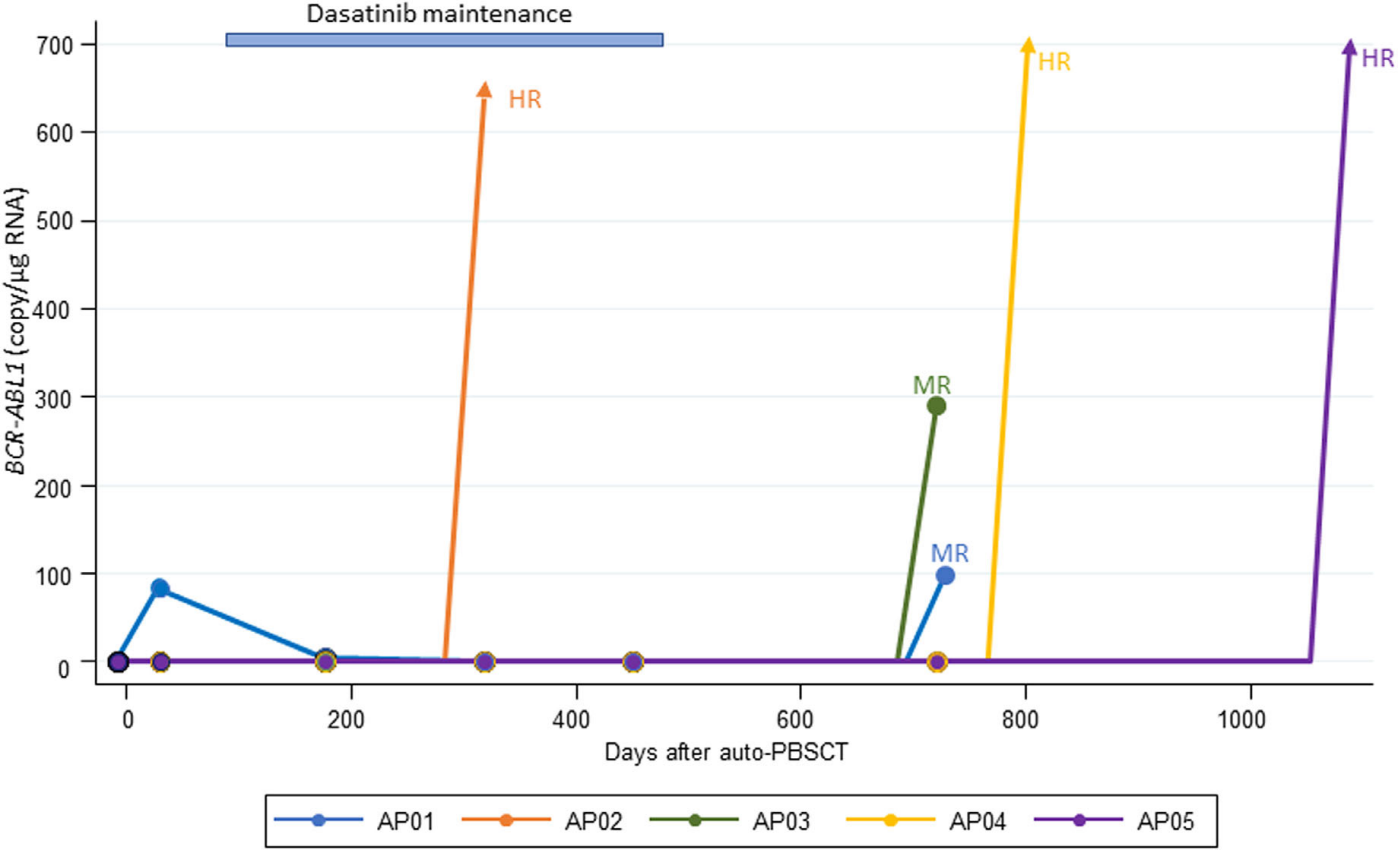
Changes in *BCR‐ABL1* copy numbers after autologous peripheral blood stem cell transplantation (auto‐PBSCT).

Although molecular relapse was observed at the checkup 30 days after auto‐PBSCT in one patient, MRD became negative again with the initiation of maintenance therapy. Molecular progressive disease was observed at a median of 731 days after auto‐PBSCT (range, 381–1088 days) (Figure 5). In 4 patients who completed 12 courses of maintenance therapy, the median time from the end of maintenance therapy to molecular progressive disease was 266 days (range, 252–611 days). Table 4 summarizes the outcomes for each patient.

**TABLE 4:** Summary of outcomes for each patient

Patient ID	AP01	AP02	AP03	AP04	AP05
*PBSCT*					
Infused CD34^+^ cells, ×10^6^/kg	3.74	4.44	2.00	3.03	2.00
Neut engraft (day)	10	9	12	14	8
Plt engraft (day)	13	21	12	14	8
RBC transfusion (unit)	2	2	4	2	4
Plt transfusion (unit)	30	50	20	60	50
*Day 100 state after PBSCT*					
Alive/dead	Alive	Alive	Alive	Alive	Alive
Molecular relapse (Y/N)	Y	N	N	N	N
Molecular progressive disease (Y/N)	N	N	N	N	N
Hematological relapse (Y/N)	N	N	N	N	N
Non‐relapsed death (Y/N)	N	N	N	N	N
*1‐year state after PBSCT*					
Alive/Dead	Alive	Alive	Alive	Alive	Alive
Molecular relapse (Y/N)	Y	N	N	N	N
Molecular progressive disease (Y/N)	N	N	N	N	N
Hematological relapse (Y/N)	N	N	N	N	N
Non‐relapsed death (Y/N)	N	N	N	N	N
*Last follow‐up*					
Last state	Alive with CR	Dead	Alive with CR	Alive with disease	Alive with disease
Molecular relapse (Y/N)	Y	Y	Y	Y	Y
Molecular progressive disease (Y/N)	Y	Y	Y	Y	Y
Hematological relapse (Y/N)	N	Y	N	Y	Y
Non‐relapsed death (Y/N)	N	N	N	N	N
PBSCT to Molecular progressive disease (days)	731	381	721	801	1088
OS (days)	1219+	1051	1519+	837+	1100+
EFS (days)	1219+	389	1519+	801	1088

Abbreviations: EFS, event‐free survival; Neut, neutrophil; OS, overall survival; PBSCT, peripheral blood stem cell transplantation; Plt, platelet; RBC, red blood cell.

### 3.6 T‐cell immunity

**FIGURE 6:**
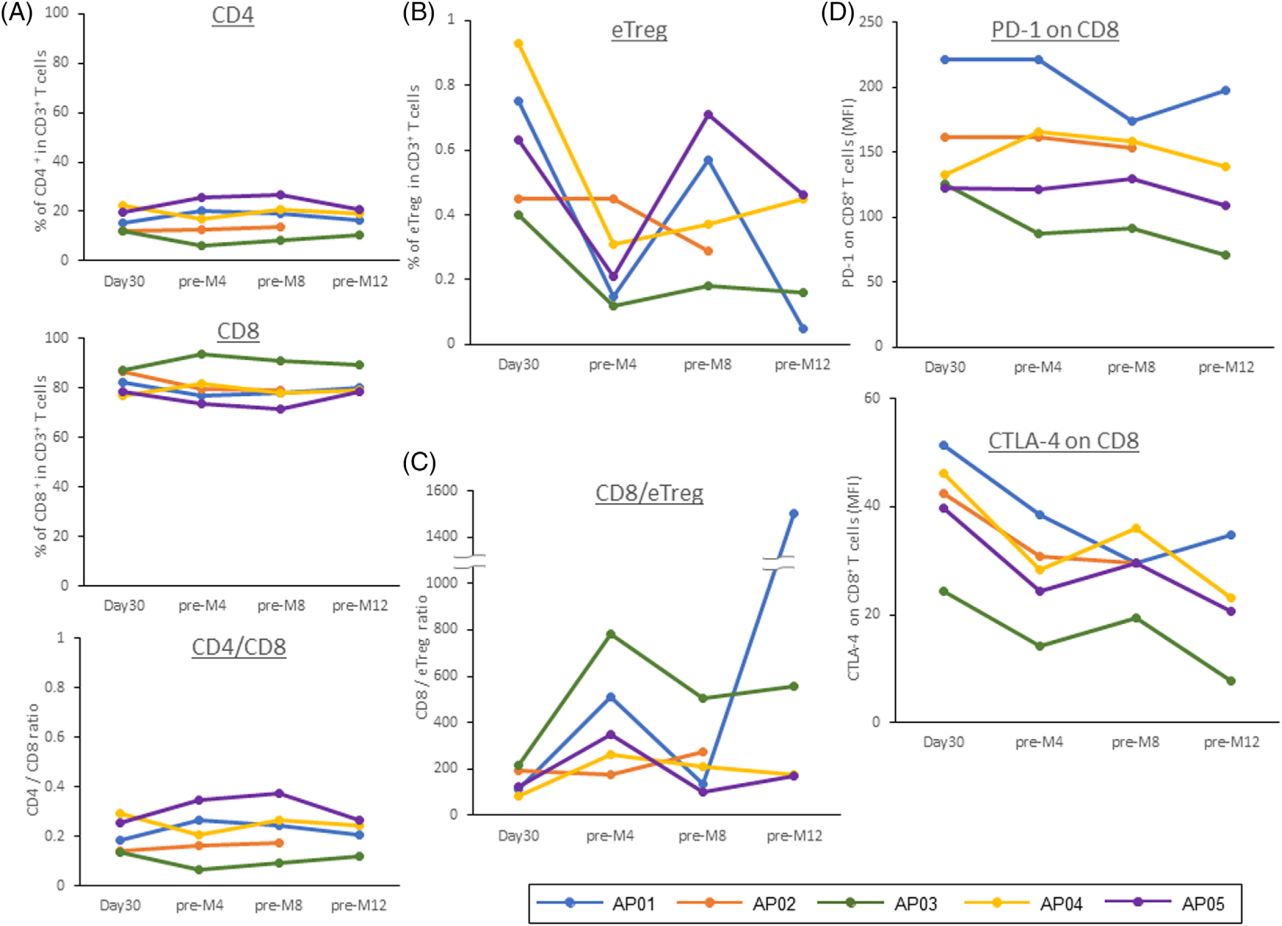
Changes in T‐cell immunity after autologous peripheral blood stem cell transplantation (auto‐PBSCT): (A) CD4^+^ and CD8^+^ cells, (B) eTreg cells, (C) CD8/eTreg ratio, (D) PD‐1 and CTLA‐4 expressions on CD8^+^ T cells.

Serial changes in peripheral T cells after auto‐PBSCTs were assessed with FCM at the designated sampling points. The percentages of CD4^+^ and CD8^+^ cells among T cells were almost constant after auto‐PBSCT, and the CR4/CD8 ratio remained low throughout the observation period (Figure 6A). On the other hand, the percentage of effector Tregs (eTreg; CD4^+^ CR45RA^−^ FOXP3^high^) cells tended to decrease once at pre‐M4 (Figure 6B). However, there was no decrease at pre‐M4 in AP‐02 patients who relapsed early after auto‐PBSCT.

The CD8/eTreg ratio was higher in the two patients who survived without hematological relapse (AP‐01 and AP‐03) and was particularly high in AP‐03, who received a lower dose of dasatinib (Figure 6C). In addition, PD‐1 and CTLA‐4 expressions on CD8^+^ T cells were relatively low in patient AP‐03 (Figure 6D).

## 4 DISCUSSION

This study prospectively evaluated the safety and efficacy of auto‐PBSCT for Ph+ALL. For PBSCH, the required number of CD34^+^ cells was collected in all cases. All patients survived for at least 100 days after auto‐PBSCT with no unexpected serious adverse events, indicating that there were no safety concerns. However, all patients eventually experienced molecular progressive disease or hematological relapse. If the goal was to develop a cure, the long‐term results were not satisfactory.

As NRM in allo‐SCT becomes higher as patient age increases, especially for patients aged 55 years or older[14], the age of eligibility for this study was set at 55 years or older. Although auto‐PBSCT is a more potent treatment than conventional chemotherapy, the fact that no treatment‐related deaths were observed suggested that the safety considerations were adequate in this study.

The 3‐year OS after allo‐SCT with reduced intensity conditioning was reported to be 64.2% in a study that analyzed Japanese registry data [15]. As for chemotherapy without allo‐SCT in CR1, the 4‐year OS of patients who achieved CMR after 3 months was reported to be 63% [16]. Although it should be evaluated carefully due to the small number of patients, the 3‐year OS of 75% in this study was not disappointing, considering that the median age was relatively high. Given that the median time from auto‐PBSCT to molecular progressive disease was as long as 731 days, it is possible that MRD became negative for a long period due to the deep molecular remission obtained by auto‐PBSCT.

This study was planned with the concept of aiming for a cure by eliminating leukemia with high‐dose chemotherapy followed by auto‐PBSCT. However, the antileukemic effect achieved by donor immunity could not be obtained, unlike allo‐SCT. Relapse after a long period since auto‐PBSCT suggested a limit to increasing the intensity of a single‐chemotherapy treatment. Recently, TKI plus blinatumomab showed excellent outcomes for de novo Ph+ALL patients [17, 18]. The efficacy of blinatumomab maintenance after allo‐SCT for B‐ALL has also been reported [19]. In addition, it has been reported that ponatinib, which is a third‐generation TKI, improves treatment results for Ph+ALL patients [20]. Long‐term use of a novel TKI and other molecular targeted drugs without allo‐SCT could be a new therapeutic strategy.

It is interesting that the patient with the earliest relapse (AP‐02) had higher eTreg cells and a lower CD8/eTreg ratio about 4 months before relapse. Increased eTreg cells may have led to early relapse by suppressing the antileukemic immune response. However, a careful interpretation of this outcome is required due to the small number of cases in this analysis. An increase in eTreg cells after autologous SCT is reportedly associated with early relapse in multiple myeloma [21] and attempts to deplete Treg cells in autologous SCT have been performed [22].

A high CD8/Treg ratio in solid tumor tissue correlates with a favorable prognosis [23]. In the present study, patients who survived without hematological relapse after auto‐PBSCT showed a higher CD8/eTreg ratio, suggesting the existence of an immune response. As for dasatinib, it has been reported that dasatinib has a potential impact on immune cells through its off‐target effects on various tyrosine kinase molecules. The proliferation of CD8^+^ T cells was reported to be inhibited by dasatinib in a dose‐dependent manner [24], which suggested high doses of dasatinib are undesirable in terms of antileukemic immunity. Moreover, eTreg cells could be selectively inhibited by low doses of imatinib and dasatinib [25]. The relatively high CD8/eTreg ratio and low expression levels of PD‐1 and CTLA‐4 on CD8^+^ T cells indicated that the selective depletion of eTreg cells was established by low‐dose dasatinib therapy in patient AP‐03 (Figure 6C,D).

In conclusion, auto‐PBSCT can be safely performed for patients with Ph+ALL. In all cases, molecular remission was sustained for more than a year after auto‐PBSCT, but disease progression was eventually observed. Long‐term treatment strategies, such as continued maintenance therapy incorporating new molecular targeted drugs, could be promising.

## AUTHOR CONTRIBUTIONS

Satoshi Nishiwaki, Isamu Sugiura, and Hitoshi Kiyoi designed the study, analyzed the data, and wrote the manuscript. Takahiko Sato, Daisuke Sugiyama, Sachiko Ito, and Hiroyoshi Nishikawa performed immunological analyses. Miki Kobayashi, Masahide Osaki, Masashi Sawa, Yoshitaka Adachi, Motohito Okabe, Shigeki Saito, Takanobu Morishita, Akio Kohno, Takahiro Nishiyama, Hiroatsu Iida, and Shingo Kurahashi registered patients and performed the protocol treatment. Yachiyo Kuwatsuka contributed to the data management and analysis of the data. All authors critically reviewed the manuscript and checked the final manuscript.

## CONFLICT OF INTEREST STATEMENT

M.S. received honoraria from Kyowa Kirin Co., Ltd., Chugai Pharmaceutical Co., Pfizer Inc., Astellas Pharma Inc., Nippon Shinyaku, Ono Pharmaceutical, MSD, Bristol‐Myers Squibb, Asahi Kasei, Novartis Pharma K.K., Eisai Co., Ltd., Otsuka Pharmaceutical Co., Ltd., Sumitomo Dainippon Pharma Co., Sanofi S.A., Takeda Pharmaceutical Co., Ltd., Celgene, Mochida Pharmaceutical Co., Ltd., Shire plc, Mundipharma K.K., AbbVie Inc., CSL Behring, SymBio, Janssen Pharmaceutical K.K., AstraZeneca plc, Daiichi Sankyo Co., and GlaxoSmithKline plc outside of this study. H.I. received research funding from Chugai Pharmaceutical Co. and honoraria from Astellas Pharma Inc., Novartis Pharma K.K., AbbVie Inc., and Janssen outside of this study. H.N. received research funding and honoraria from Ono Pharmaceutical, MSD, Bristol‐Myers Squibb, and Chugai Pharmaceutical, and research funding from Taiho Pharmaceutical, Daiichi Sankyo, Kyowa Kirin Co., Ltd., Zenyaku Kogyo, BioPharma Inc., Debiopharm, Asahi Kasei, Sysmex, FUJIFILM Corporation, SRL, Astellas Pharma Inc., Sumitomo Dainippon Pharma Co., and BD Japan outside of this study. H.K. received research funding from FUJIFILM Corporation, Kyowa Hakko Kirin Co., Ltd., Bristol‐Myers Squibb, Otsuka Pharmaceutical Co., Ltd., Perseus Proteomics Inc., Daiichi Sankyo Co., Ltd., AbbVie Inc., CURED, Inc., Astellas Pharma Inc., Chugai Pharmaceutical Co., Ltd., Zenyaku Kogyo Co., Ltd., Nippon Shinyaku Co., Ltd., Eisai Co., Ltd., Takeda Pharmaceutical Co., Ltd., Sumitomo Dainippon Pharma Co., Ltd., Novartis Pharma K.K., and Sanofi K.K., and honoraria from Astellas Pharma Inc., AbbVie Inc., Chugai Pharmaceutical Co., Ltd., and Novartis Pharma K.K. outside of this study. The remaining authors declare no competing financial interests.

## ETHICS STATEMENT

This study was approved by the institutional review board of each participating hospital.

## PATIENT CONSENT STATEMENT

All participants gave their informed written consent to participate in this study.

## CLINICAL TRIAL REGISTRATION

UMIN000026445.

## Supporting information

Supporting Information

## Data Availability

The data that support the findings of this study are available from the corresponding author upon reasonable request.

## References

[1] Sehn LH , Salles G . Diffuse large B‐cell lymphoma. N Engl J Med. 2021;384(9):842–58. 33657296 10.1056/NEJMra2027612 PMC8377611

[2] Cowan AJ , Green DJ , Kwok M , Lee S , Coffey DG , Holmberg LA , et al. Diagnosis and management of multiple myeloma: a review. JAMA. 2022;327(5):464–77. 35103762 10.1001/jama.2022.0003

[3] Pang A , Huo Y , Shen B , Zheng Y , Jiang E , Feng S , et al. Optimizing autologous hematopoietic stem cell transplantation for acute leukemia. Stem Cells Transl Med. 2021;10(Suppl 2):S75–84. 34724713 10.1002/sctm.21-0176 PMC8560201

[4] Kim K , Jabbour E , Short NJ , Kebriaei P , Kantarjian H , Ravandi F . Current approaches to Philadelphia chromosome‐positive B‐cell lineage acute lymphoblastic leukemia: role of tyrosine kinase inhibitor and stem cell transplant. Curr Oncol Rep. 2021;23(8):95. 34125415 10.1007/s11912-021-01086-y PMC11781348

[5] Yanada M , Takeuchi J , Sugiura I , Akiyama H , Usui N , Yagasaki F , et al. High complete remission rate and promising outcome by combination of imatinib and chemotherapy for newly diagnosed BCR‐ABL‐positive acute lymphoblastic leukemia: a phase II study by the Japan Adult Leukemia Study Group. J Clin Oncol. 2006;24(3):460–6. 16344315 10.1200/JCO.2005.03.2177

[6] Wetzler M , Watson D , Stock W , Koval G , Mulkey FA , Hoke EE , et al. Autologous transplantation for Philadelphia chromosome‐positive acute lymphoblastic leukemia achieves outcomes similar to allogeneic transplantation: results of CALGB Study 10001 (Alliance). Haematologica. 2014;99(1):111–5. 24077846 10.3324/haematol.2013.085811 PMC4007937

[7] Chalandon Y , Thomas X , Hayette S , Cayuela JM , Abbal C , Huguet F , et al. Randomized study of reduced‐intensity chemotherapy combined with imatinib in adults with Ph‐positive acute lymphoblastic leukemia. Blood. 2015;125(24):3711–9. 25878120 10.1182/blood-2015-02-627935

[8] Giebel S , Labopin M , Potter M , Poire X , Sengeloev H , Socie G , et al. Comparable results of autologous and allogeneic haematopoietic stem cell transplantation for adults with Philadelphia‐positive acute lymphoblastic leukaemia in first complete molecular remission: an analysis by the Acute Leukemia Working Party of the EBMT. Eur J Cancer. 2018;96:73–81. 29679774 10.1016/j.ejca.2018.03.018

[9] Nishiwaki S , Sugiura I , Miyata Y , Saito S , Sawa M , Nishida T , et al. Efficacy and safety of autologous peripheral blood stem cell transplantation for Philadelphia chromosome‐positive acute lymphoblastic leukemia: a study protocol for a multicenter exploratory prospective study (Auto‐Ph17 study). Medicine (Baltimore). 2017;96(52):e9568. 29384978 10.1097/MD.0000000000009568 PMC6393033

[10] Kaplan EL , Meier P . Nonparametric estimation from incomplete observations. J Am Stat Assoc. 1958;53:457–81.

[11] Gooley TA , Leisenring W , Crowley J , Storer BE . Estimation of failure probabilities in the presence of competing risks: new representations of old estimators. Stat Med. 1999;18(6):695–706. 10204198 10.1002/(sici)1097-0258(19990330)18:6<695::aid-sim60>3.0.co;2-o

[12] Scrucca L , Santucci A , Aversa F . Competing risk analysis using R: an easy guide for clinicians. Bone Marrow Transplant. 2007;40(4):381–7. 17563735 10.1038/sj.bmt.1705727

[13] Kanda Y . Investigation of the freely available easy‐to‐use software ‘EZR’ for medical statistics. Bone Marrow Transplant. 2013;48(3):452–8. 23208313 10.1038/bmt.2012.244 PMC3590441

[14] Nishiwaki S , Imai K , Mizuta S , Kanamori H , Ohashi K , Fukuda T , et al. Impact of MRD and TKI on allogeneic hematopoietic cell transplantation for Ph+ALL: a study from the adult ALL WG of the JSHCT. Bone Marrow Transplant. 2016;51(1):43–50. 26389833 10.1038/bmt.2015.217

[15] Akahoshi Y , Nishiwaki S , Arai Y , Harada K , Najima Y , Kanda Y , et al. Reduced‐intensity conditioning is a reasonable alternative for Philadelphia chromosome‐positive acute lymphoblastic leukemia among elderly patients who have achieved negative minimal residual disease: a report from the Adult Acute Lymphoblastic Leukemia Working Group of the JSHCT. Bone Marrow Transplant. 2020;55(7):1317–25. 32447350 10.1038/s41409-020-0951-0

[16] Short NJ , Jabbour E , Sasaki K , Patel K , O'Brien SM , Cortes JE , et al. Impact of complete molecular response on survival in patients with Philadelphia chromosome‐positive acute lymphoblastic leukemia. Blood. 2016;28(4):504–7. 10.1182/blood-2016-03-707562 PMC4965905 27235138

[17] Foà R , Bassan R , Vitale A , Elia L , Piciocchi A , Puzzolo MC , et al. Dasatinib‐Blinatumomab for Ph‐positive acute lymphoblastic leukemia in adults. N Engl J Med. 2020;383(17):1613–23. 33085860 10.1056/NEJMoa2016272

[18] Short N , Kantarjian H , Konopleva M , Jain N , Huang X , Ravandi F , et al. Combination of ponatinib and blinatumomab in Philadelphia chromosome‐positive acute lymphoblastic leukemia: early results from a phase II study. J Clin Oncol. 2021;39(Suppl 15):7001.

[19] Gaballa MR , Banerjee P , Milton DR , Jiang X , Ganesh C , Khazal S , et al. Blinatumomab maintenance after allogeneic hematopoietic cell transplantation for B‐lineage acute lymphoblastic leukemia. Blood. 2022;139(12):1908–19. 34914826 10.1182/blood.2021013290 PMC8952188

[20] Jabbour E , Kantarjian H , Ravandi F , Thomas D , Huang X , Faderl S , et al. Combination of hyper‐CVAD with ponatinib as first‐line therapy for patients with Philadelphia chromosome‐positive acute lymphoblastic leukaemia: a single‐centre, phase 2 study. Lancet Oncol. 2015;16(15):1547–55. 26432046 10.1016/S1470-2045(15)00207-7 PMC4816046

[21] Batorov EV , Tikhonova MA , Pronkina NV , Kryuchkova IV , Sergeevicheva VV , Sizikova SA , et al. Increased circulating CD4(+)FOXP3(+) T cells associate with early relapse following autologous hematopoietic stem cell transplantation in multiple myeloma patients. Oncotarget. 2018;9(43):27305–17. 29930767 10.18632/oncotarget.25553 PMC6007464

[22] Derman BA , Zha Y , Zimmerman TM , Malloy R , Jakubowiak A , Bishop MR , et al. Regulatory T‐cell depletion in the setting of autologous stem cell transplantation for multiple myeloma: pilot study. J Immunother Cancer. 2020;8(1):e000286. 31940591 10.1136/jitc-2019-000286 PMC7057425

[23] Sato E , Olson SH , Ahn J , Bundy B , Nishikawa H , Qian F , et al. Intraepithelial CD8+ tumor‐infiltrating lymphocytes and a high CD8+/regulatory T cell ratio are associated with favorable prognosis in ovarian cancer. Proc Natl Acad Sci USA. 2005;102(51):18538–43. 16344461 10.1073/pnas.0509182102 PMC1311741

[24] Fei F , Yu Y , Schmitt A , Rojewski MT , Chen B , Greiner J , et al. Dasatinib exerts an immunosuppressive effect on CD8+ T cells specific for viral and leukemia antigens. Exp Hematol. 2008;36(10):1297–308. 18619726 10.1016/j.exphem.2008.05.002

[25] Tanaka A , Nishikawa H , Noguchi S , Sugiyama D , Morikawa H , Takeuchi Y , et al. Tyrosine kinase inhibitor imatinib augments tumor immunity by depleting effector regulatory T cells. J Exp Med. 2020;217(2):e20191009. 31704808 10.1084/jem.20191009 PMC7041710

