# Supplementary material for: Autologous peripheral blood stem cell transplantation for Philadelphia chromosome‐positive acute lymphoblastic leukemia is safe but poses challenges for long‐term maintenance of molecular remission: Results of the Auto‐Ph17 study
Source: EJHaem. 2023 Mar 20;4(2):358–69. doi: 10.1002/jha2.677 (PMC10188459; doi:10.1002/jha2.677)
Supplement: Supplementary file 1 — Supporting Information [file JHA2-4-358-s001.doc]

**TABLE S1.** Summary of antibodies used in flow cytometry analyses.

| **Tag** | **Molecule** | **Clone** | **Company** |
| --- | --- | --- | --- |
| BUV496 | Human CD8 | HIT8a | BD Biosciences |
| BUV615 | Human CD4 | SK3 | BD Biosciences |
| BUV661 | Human LAG-3 | T47-530 | BD Biosciences |
| BV421 | Human CD279 (PD-1) | MIH4 | BD Biosciences |
| BV480 | Human TIM-3 | 7D3 | BD Biosciences |
| BV650 | Human CD3 | SK7 | BD Biosciences |
| BV711 | Human CD45RA | HI100 | BD Biosciences |
| PE | Human FOXP3 | 236A/E7 | Thermo Fisher Scientific |
| PE-CF594 | Human CD152 (CTLA-4) | BNI3 | BD Biosciences |
| PE-Cy7 | Human CD197 (CCR7) | G043H7 | Biolegend |
| APC-R700 | Human HLA-DR | G46-6 | BD Biosciences |
